# Migrant and ethnic minorities at higher risk of COVID-19 severe outcomes? A systematic review: Elena Mazzalai

**DOI:** 10.1093/eurpub/ckac131.092

**Published:** 2022-10-25

**Authors:** E Mazzalai, D Giannini, ME Tosti, A Jaljaa, S Caminada, F Turatto, C De Marchi, A Gatta, G Marchetti, M Marceca

**Affiliations:** 1 Department of Public Health and Infectious Diseases, Sapienza University of Rome, Rome, Italy; 2 National Centre for Global Health, National Health Institute, Rome, Italy

## Abstract

**Background:**

The Covid-19 pandemic has had a recognised impact in widening health inequalities, both between and within countries, with a major impact on socially disadvantaged population groups such as Migrants and Ethnic Minorities (MEMs). While there is growing evidence on the matter worldwide, data specific to the WHO European Region is scarce. The issue, however, is pressing, since it is estimated that almost 10% of the population living in the WHO European Region is made up of migrants. The aim of the study is to investigate the impact of Covid-19 on MEMs compared to the general population in terms of serious outcomes.

**Methods:**

We conducted a systematic review collecting studies on the impact of Covid-19 on MEMs compared to the general population in the WHO European Region regarding hospitalisation, intensive care unit (ICU) admission and mortality, published between 01/01/2020 and 19/03/2021. Fourteen researchers were involved in selection, study quality assessment, data extraction and analysis.

**Results:**

Of the 82 studies included, 15 of the 16 regarding hospitalisation for Covid-19 reported an increased risk for MEMs compared to the white and/or native population and 22 out of the 28 studies focusing on the ICU admission rates found an increased risk for MEMs. Among the 65 studies on mortality, 43 report a higher risk for MEMs. 82% of the studies were conducted in the UK.

**Conclusions:**

These findings highlight the disproportionate impact of Covid-19 on MEMs population, with an increased risk of all the adverse outcomes taken into consideration. Social determinants of health are among the main factors involved in the genesis of health inequalities: a disadvantaged socio-economic status, a framework of structural racism and asymmetric access to healthcare are linked to increased susceptibility to the consequences of Covid-19. These findings underline the need for policy-makers to consider the socio-economic barriers when designing health promotion plans.

**Key messages:**

• The combination of disadvantage socioeconomic conditions with COVID-19 transmission characteristics put migrants and ethnic minorities at a higher risk of facing sever health outcomes.

• The amount of evidence on the inequal impact of COVID-19 on migrants and ethnic minorities produced by European countries is poor. This gap must be filled to develop effective health promotion plans.

